# Simultaneous High-Performance Recovery and Extended Acid-Catalyzed Hydrolysis of Oleuropein and Flavonoid Glycosides of Olive (*Olea europaea*) Leaves: Hydrothermal versus Ethanol Organosolv Treatment

**DOI:** 10.3390/ijms25147820

**Published:** 2024-07-17

**Authors:** Hela Refai, Feyrouz Derwiche, Spyros Grigorakis, Dimitris P. Makris

**Affiliations:** 1 Department of Food Quality & Chemistry of Natural Products, Mediterranean Agronomic Institute of Chania (M.A.I.Ch.), International Centre for Advanced Mediterranean Agronomic Studies (CIHEAM), P.O. Box 85, 73100 Chania, Greece; hela.refai@enis.tn (H.R.); feyrouzderwiche@gmail.com (F.D.); grigorakis@maich.gr (S.G.); 2 Green Processes & Biorefinery Group, Department of Food Science & Nutrition, School of Agricultural Sciences, University of Thessaly, N. Temponera Street, 43100 Karditsa, Greece

**Keywords:** acid hydrolysis, antioxidants, olive leaves, oxalic acid, polyphenols

## Abstract

Olive leaves (OLLs) are an exceptional bioresource of natural polyphenols with proven antioxidant activity, yet the applicability of OLL extracts is constrained by the relatively high polarity of the major polyphenols, which occur as glycosides. To overcome this limitation, OLLs were subjected to both hydrothermal and ethanol organosolv treatments, fostered by acid catalysis to solicit in parallel increased polyphenol recovery and polyphenol modification into simpler, lower-polarity substances. After an initial screening of natural organic acids, oxalic acid (OxAc) was found to be the highest-performing catalyst. The extraction behavior using OxAc-catalyzed hydrothermal and ethanol organosolv treatments was appraised using kinetics, while treatment optimization was accomplished by deploying response-surface methodology. The comparative assessment of the composition extracts produced under optimal conditions of residence time and temperature was performed with liquid chromatography–tandem mass spectrometry and revealed that OLLs treated with 50% ethanol/1.5% HCl suffered extensive oleuropein and flavone glycoside hydrolysis, affording almost 23.4 mg hydroxytyrosol and 2 mg luteolin per g dry weight. On the other hand, hydrothermal treatment with 5% OxAc provided 20.2 and 0.12 mg of hydroxytyrosol and luteolin, respectively. Apigenin was in all cases a minor extract constituent. The study presented herein demonstrated for the first time the usefulness of using a natural, food-grade organic acid to perform such a task, yet further investigation is needed to maximize the desired effect.

## 1. Introduction

In recent years, consumer concerns about the consequences of dietary habits on health status have pushed the food industry to increase the production of foods with enhanced bioactivities, with a parallel demand for new sources of functional compounds. A promising pathway towards this direction is the harnessing of abundant agri-food waste bioresources, which could provide a bewildering diversity of innovative constituents [1,2]. Based on contemporary biorefinery approaches, through the recovery of bioactive phytochemicals from residual agricultural material or food processing by-products, sustainable and state-of-the-art food systems may be established, reducing the cost of waste management and treatment, and offer innovative reutilization techniques in the food chain [3,4].

The Mediterranean basin is characterized by the extended cultivation of plants such as vines (*Vitis vinifera*) and olive trees (*Olea europaea*), whose fruit is regularly processed for producing a variety of food commodities [5]. With reference to the latter, a great part of olive crops is destined for the production of olive oil, with the Mediterranean countries providing almost 98% of the global olive oil production [6]. Inevitably, olive cultivation and processing generate an enormous amount of waste, composed principally of pruning residues, olive leaves, and olive mill waste waters. The management and handling of these side streams is of utmost importance in establishing sustainable manufacturing strategies, but also in diminishing environmental aggravation.

Pruning alone may be responsible for an annual production of 25 kg of waste per olive tree, which embraces branches and leaves. This is a major source of olive leaves, yet a considerable amount is also discarded during washing of the olive drupes, which takes place at the beginning of the olive oil production chain. This may represent roughly 8% to 10% of the total olive weight subjected to milling [7]. Olive leaves (OLLs) are regarded as a precious bioresource of high value-added substances, since they contain a spectrum of polyphenolic metabolites, the majority of which is the secoiridoid oleuropein, and flavone (mainly luteolin) glycosides [8]. A significant number of studies have clearly showcased the biological properties of olive leaf extracts, such as antioxidant, antimicrobial, anti-inflammatory, cytotoxic, and anti-hypertensive activities [9,10], which have been largely ascribed to their polyphenolic composition. Thus, although various methodologies have been proposed for the effective olive leaf management [11], polyphenol recovery through solid–liquid extraction holds as the most prominent process for olive tree leaf valorization [12]. On this ground, numerous technologies have been developed to provide extracts with increased polyphenol recovery and antioxidant activity [13,14].

The concept of utilizing plant extracts to fortify foods is particularly intriguing, considering that plant polyphenols are both functional constituents and antioxidant additives. On the other hand, a wide range of polyphenols occur in plants tissues, including plant food wastes, under glycosylated forms, which renders them of relatively high polarity. Thus, the incorporation of extracts enriched in such compounds into lipid substrates would be rather impractical and/or problematic, making the search for suitable candidates a challenging prospect. Recently, it was revealed that the treatment/extraction of onion solid wastes with citric acid-based natural deep eutectic solvents (NADESs) may be an effective means of converting polar flavonol (quercetin) glycosides into the more lipophilic aglycone, at the same time enhancing the antioxidant activity of the extracts obtained [15]. A follow-up study also demonstrated that such a task may be achieved with oxalic acid-based DES and, upon appropriate time/temperature regulation, quercetin-enriched extracts with increased antioxidant activity could be generated [16].

Based on this evidence, the work presented herein targeted at the olive leaf polyphenol modification (hydrolysis), in order to produce hydroxytyrosol- and flavone-enriched extracts with enhanced antioxidant activity. To this end, hydrothermal and ethanol-organosolv treatments were tested, to establish the most favorable conditions, while a natural organic acid (oxalic acid) was examined as an acid catalyst to attain high hydrolysis/extraction yields. Hydrochloric acid was also tested along with oxalic acid, to better illustrate oxalic acid’s potency to perform polyphenol glycoconjugate hydrolysis. As far as the authors are aware, such a study has never been reported for olive leaf processing, and it constitutes a new endeavor in valorizing this abundant agri-food waste for producing high value-added chemicals.

## 2. Results and Discussion

### 2.1. Effect of Acid Addition

**Figure 1:**
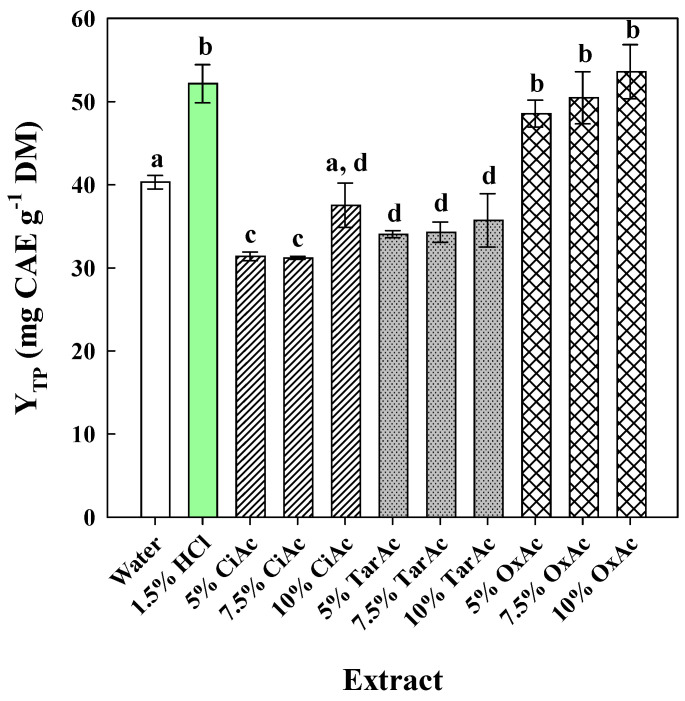
Bar plot depicting the effect of various acids and concentrations on the polyphenol recovery from OLLs. Extractions were accomplished at 90 °C, for 180 min. Bars assigned with different letters (a–d) represent statistically different values (*p* < 0.05).

To investigate the effect of acid on the total polyphenol recovery from OLLs, a series of aqueous solutions with various concentrations of natural organic acids, including citric acid (CiAc), tartaric acid (TarAc), and oxalic acid (OxAc), were tested (Figure 1), along with pure water and 1.5% HCl, which served as control treatments. The selection of these acids was based on their relatively low p*K*_a_ (first dissociation) compared to other commonly encountered ones, i.e., acetic acid and lactic acid.

The addition of CiAc at any concentration tested was unfavorable in increasing Y_TP_, as revealed from the comparison with pure water (*p* < 0.05). The same phenomenon was observed for TarAc too, whose addition resulted in a significantly lower Y_TP_ compared to pure water (*p* < 0.05). On the contrary, OxAc at any concentration tested contributed to attaining a Y_TP_ as high as that of 1.5% HCl (*p* < 0.05).

The use of aqueous organic acid media for effective polyphenol extraction has been examined by earlier studies [17]. The importance of performing polyphenol extraction in the presence of acid lies in the ability of certain acids to boost recovery yield. Such an effect has been demonstrated for materials such as onion solid wastes [18] and grape pomace [19]. In some cases, the effect was shown to be concentration-dependent [20], although the outcome of other examinations on the effect of organic acid have been controversial [21]. The actual effect that the acid may exert on the polyphenol recovery process could be rather multifaceted. First, low pH imparted by the presence of the acid may have a protected action on polyphenols, since many of them may be prone to oxidation at pH values closer to neutrality. Such a behavior has been demonstrated for polyphenols from various sources [22,23]. Therefore, maintaining a low pH, especially at relatively high processing temperatures, could be crucial to minimizing polyphenol losses.

However, an issue of high importance is the disruption of plant cell integrity, to enable the release of intracellular metabolites (polyphenols) and their entrainment into the liquid phase (solvent). The disintegration of cell walls is a key event in this regard [24,25], and this can be accomplished by disorganizing the recalcitrant lignin–hemicellulose–cellulose matrix, but also pectin, which are the major cell wall structural networks. The use of organic acids may greatly contribute to cell wall polysaccharide hydrolysis, resulting in extended cell wall breakdown. The comparative examination of organic acids as catalysts demonstrated that hydrothermal pretreatment of corn stover to produce xylose (a hemicellulose hydrolysis product) was more effective with sulfuric and oxalic acids than with acetic, citric, and tartaric acids [26]. This ability was linked to the p*K*_a_ of the acids tested. In addition, lignin cleavage has also been accomplished employing aqueous oxalic acid solutions [27]. Several other studies have also shown that biomass treatments involving oxalic acid as a catalyst may be highly effective in hydrolyzing hemicellulose and/or releasing lignin, hence contributing to untangling the lignocellulosic complex [28,29,30]. Thus, the results given in Figure 1 might reflect the ability of oxalic acid to disorganize cell walls and promote the liberation of intracellular polyphenols, thereby increasing polyphenol recovery.

### 2.2. Ethanol Effect

**Figure 2:**
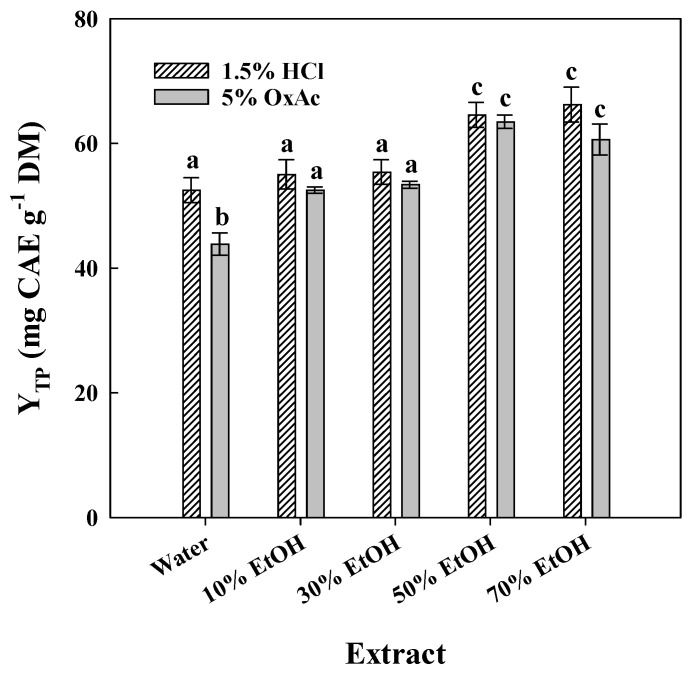
Bar plot presenting the effect of water/ethanol proportion (*v*/*v*) on the polyphenol recovery from OLLs, using 1.5% HCl or 5% oxalic acid as acid catalysts. Bars denoted with different letters (a–c) are statistically different (*p* < 0.05).

From the screening of acids, it was concluded that aqueous 5% OxAc was equally effective to 1.5% HCl with respect to polyphenol recovery. Thus, these two solvent systems were further considered to examine the effect of ethanol. The ethanol concentration range tested was from 10 to 70% (*v*/*v*), in compliance with previous investigations which suggested optimum ethanol levels for OLL polyphenol extraction to be around 50–70% [1,31,32,33]. Ethanol levels of 10 and 30% had a negligible effect compared to water, in solvents containing 1.5% HCl (Figure 2).

By contrast, the combination of either 10 or 30% ethanol with 5% OxAc significantly enhanced the total polyphenol yield (*p* < 0.05). A further increase in the ethanol level to 50% boosted polyphenol recovery even higher, irrespective of the catalyst used (HCl or OxAc). However, the increase in ethanol concentration from 50 to 70% afforded virtually equal yields (*p* > 0.05). On this ground, the ideal solvent system was deemed to be 50% aqueous ethanol containing 5% OxAc.

### 2.3. Acid and Ethanol Effects on Extraction Kinetics

**Figure 3:**
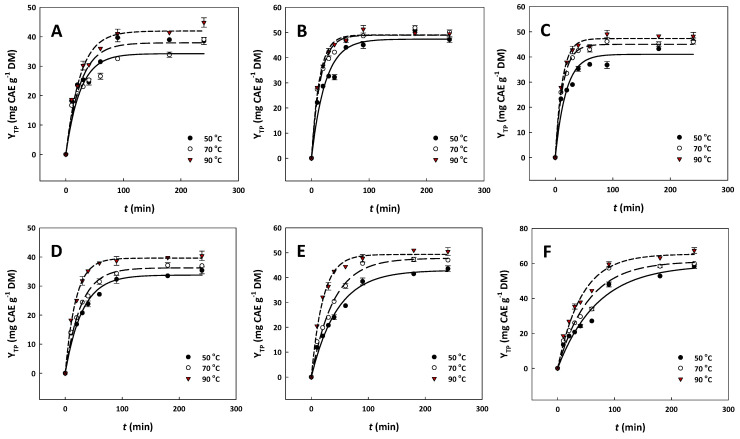
Polyphenol extraction kinetics during treatment of OLLs with various solvent systems: (**A**) water; (**B**) 1.5% aqueous HCl; (**C**) 5% aqueous oxalic acid; (**D**) 50% ethanol; (**E**) 50% ethanol/1.5% HCl; (**F**) 50% ethanol/5% oxalic acid. Bars represent the standard deviation.

To better clarify the role of acid and ethanol in the extractability of OLL polyphenols, the kinetics was traced over a temperature range varying from 50 to 90 °C (Figure 3). In all cases investigated, the best model fitting was first-order kinetics, and on this ground the extraction rate constant, *k*, and the yield in total polyphenols at saturation, Y_TP(s)_, were determined (Table 1).

With neat water, the maximum extraction rate (0.0405 min^−1^) was recorded at 90 °C, and the Y_TP(s)_ was found to be 41.97 mg CAE g^−1^ DM (Figure 3A). The temperature rising from 50 to 90 °C had a positive effect on *k*, as it increased from 0.0398 to 0.0405 min^−1^, yet the particularly low *E*_a_ determined (0.52 kJ mol^−1^) clearly suggested a very weak dependence of the polyphenol extraction rate on temperature. Considering that the Y_TP(s)_ attained at 50 °C showed no statistical difference with the one obtained at 90 °C (*p* < 0.05), it could be argued that treatment with water might reach a maximum yield at 50 °C, and beyond this point temperature increase contributed neither to accelerating polyphenol leaching nor to boosting the polyphenol recovery yield.

On the other hand, the addition of 1.5% HCl was shown to significantly favor both *k* and Y_TP(s)_, and at 90 °C the corresponding values were 0.0731 min^−1^ and 49.06 mg CAE g^−1^ DM (Figure 3B). This finding clearly pointed to a favorable effect of HCl, regarding both the polyphenol extraction rate and the recovery yield. The effect of 5% OxAc was similar, providing even an higher *k* (0.0827 min^−1^) and a comparable Y_TP(s)_ of 47.33 mg CAE g^−1^ DM (*p* < 0.05), at 90 °C (Figure 3C). Thus, for the hydrothermal treatment, acidification unequivocally had a positive impact on polyphenol recovery from OLLs. Furthermore, the energetic barriers for the treatments with 1.5% HCl and 5% OxAc (5.88 and 5.02 kJ mol^−1^, respectively) were virtually equal, revealing that the treatment with a natural, non-corrosive acid, such as OA, may be as effective as the one performed with HCl.

The organosolv treatment with 50% ethanol afforded, at 90 °C, practically the same Y_TP(s)_ with the hydrothermal treatment with neat water but exhibited a higher *k* (0.0544 min^−1^) and it had a significantly higher barrier (19.80 kJ mol^−1^). These results indicated that, with the organosolv treatment, the polyphenol recovery rate had a greater dependence on temperature, yet the Y_TP(s)_ reached the same maximum. When treatment was performed with 50% ethanol containing 1.5% HCl, it was observed that the recovery rate was significantly slowed down to 0.0490 min^−1^ at 90 °C, and the Y_TP(s)_ increased to 49.32 mg CAE g^−1^ DM, while there was also a significant increase in *E*_a_, which equaled 32.81 kJ mol^−1^ (Figure 3E). This phenomenon manifested a much stronger dependence of the treatment on temperature, and a higher efficacy in polyphenol recovery. On the contrary, the incorporation of 5% OxAc in 50% ethanol provoked a significant drop in the barrier at 12.84 kJ mol^−1^ and displayed the lowest *k* at any temperature tested, compared to all other treatments. On the other hand, the treatment with 50% ethanol/5% OxAc was the most efficacious regarding Y_TP(s)_, giving a level of 65.36 mg CAE g^−1^ DM (Figure 3F).

The importance of aqueous strong acid (sulfuric acid) in OLL polyphenol recovery has been reported in an earlier study [34]. Considering that outcome, and based on the above-mentioned, the treatment with neat water and 50% ethanol provided the same Y_TP(s)_, which essentially reached an upper limit. Therefore, it could be argued that, irrespective of the solvent, polyphenol recovery yield could not exceed this limit without acid addition. The addition of HCl in either water or 50% ethanol boosted Y_TP(s)_, which once again did not go beyond a certain level. On the other hand, the addition of 5% OxAc in either water or 50% ethanol favored the obtaining of a higher Y_TP(s)_, but it also gave a 33% higher Y_TP(s)_ compared to 50% ethanol/1.5% HCl at 90 °C. This outcome highlighted the stronger effect of OxAc, compared to HCl.

The interpretation of the phenomena observed might be mostly related to the effect of the acids, as analyzed in Section 2.1. With non-acidified solvents (water, 50% ethanol), polyphenol recovery may be achieved with the washing of polyphenol molecules from the external surface of the solid particles (OLLs), and penetration of the solvent into the particles, dissolution of the solute (polyphenols), and entrainment of the solute into the liquid phase, outside the particle. Such a process may advance to some extent, but the integrity of the cells is not acted upon. In the presence of any acid (HCl, OxAc), cell wall disintegration may occur, facilitating solvent penetration to the interior of the cells, dissolution, and solute entrainment. Such an effect would both accelerate polyphenol recovery rate and increase polyphenol recovery yield, presumably due to higher internal diffusion, which is the rate-limiting step in the extraction process [35]. In the case of the hydrothermal treatment, this assumption is supported by the higher *k* and Y_TP(s)_ determined for 1.5% HCl and 5% OxAc, compared to neat water.

However, the incorporation of any acid (HCl, OxAc) in 50% hydroethanolic solutions significantly reduced the polyphenol extraction rate compared to 50% ethanol, a phenomenon that was more pronounced with OxAc. Nevertheless, the addition of OxAc contributed to achieving the highest Y_TP(s)_, and in this regard, 50% ethanol/5% OxAc was the highest-performing system. The assumption that could be made to explain such a behavior of acid/ethanol combination is that acid dissociation in hydroethanolic solvents is much weaker compared to aqueous media [36,37], and this could hinder hydrolytic reactions that would lead to cell wall deconstruction and an increased extraction rate, as discussed above. In this case, cell wall breakdown would probably require harsher conditions (e.g., higher temperatures), which might explain why organosolv treatments required a higher *E*_a_ compared to the hydrothermal ones (Table 2). The fact that the combination of 50% ethanol with 5% OxAc boosted Y_TP(s)_ might indicate that OxAc, which remained largely undissociated in 50% ethanol, acted possibly as a co-solvent, thus further reducing water polarity and facilitating polyphenol dissolution. This would explain the significantly lower *E*_a_ found for the treatment carried out with 50% ethanol/5% OxAc, compared to that performed with 50% ethanol/1.5% HCl. Such a theory remains to be elucidated.

### 2.4. Response-Surface Methodology and Treatment Optimization

**Figure 4:**
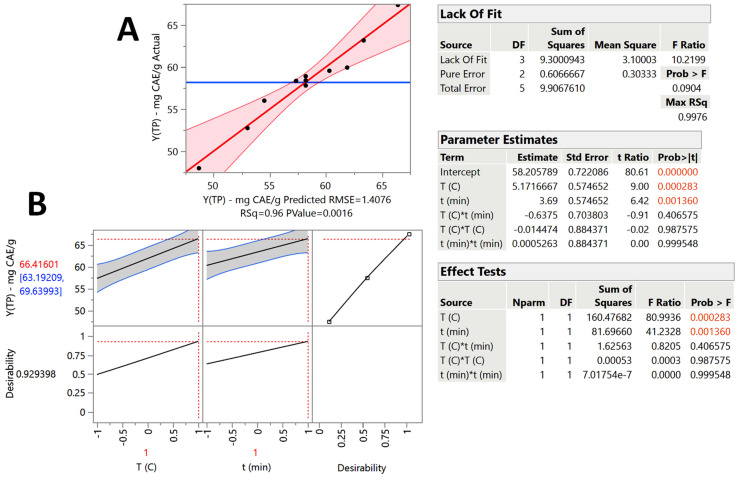
(**A**) Correlation between the actual and the predicted values of the response (Y_TP_), as determined by response-surface methodology, implemented to model the effect of residence time (*t*) and temperature (*T*) on the polyphenol recovery from OLLs. (**B**) Desirability plot showing the codified values of the optimum conditions (*t*, *T*) and the optimum predicted response (Y_TP_) under these conditions. Inset tables display the statistical information obtained after performing lack-of-fit and ANOVA tests. Colored values are statistically significant (*p* < 0.05).

The kinetic investigation clearly revealed that the system 50% ethanol/5% OxAc was highly efficacious in recovering OLL polyphenols, and thus this solvent was selected to carry out treatment optimization, based on response-surface methodology. The experimental design deployed aimed to assess the effects of the two critical independent variables, *T* and *t*, on Y_TP_, which was chosen as the response, but also to detect possible synergistic (cross) functions between the two independent variables. The treatment modeling validity was evaluated considering the results from the analysis of variance (ANOVA) and lack-of-fit tests (Figure 4, inset tables).

For every design point, the predicted response (Y_TP_) was calculated, and it is given in Table 2, along with the actual (measured) values. The correlation between the measured and predicted response values may be seen in Figure 4A. Based on the “Parameter Estimates” and “Effect Tests” (inset tables in Figure 4), the non-significant terms were excluded from the model (equation) derived, which was as follows:

Y_TP_ = 58.21 + 5.17X_1_ + 3.69X_2_ (R^2^ = 0.96, *p* = 0.0016) (1)

**Figure 5:**
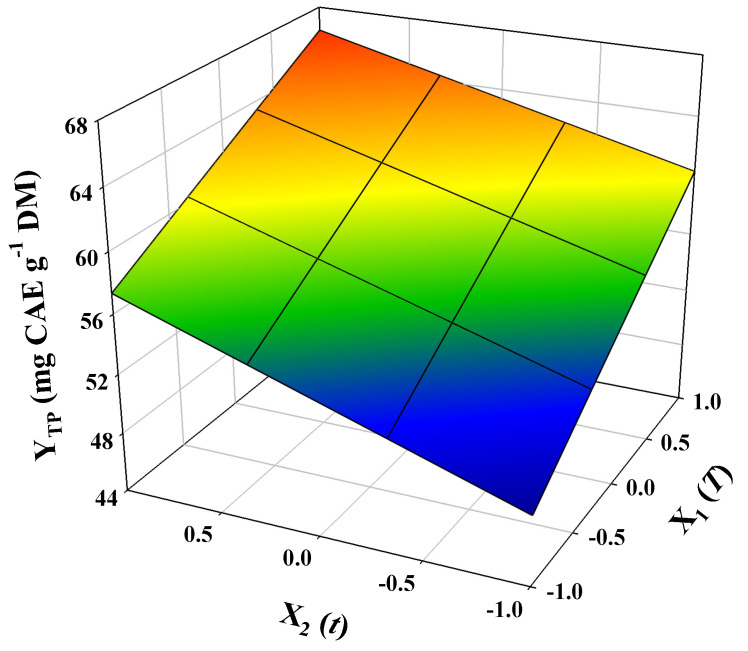
Graphical 3D representation of the effect of the independent variables (*t*, *T*) on the response (Y_TP_), as revealed by deploying response-surface methodology.

Considering the overall model R^2^ and the *p* value significant for lack-of-fit (confidence interval of at least 95%), it was affirmed that the model produced a very satisfactory fitting to the experimental data. The simultaneous effect of the independent variables on the response was portrayed by a 3D diagram (Figure 5). The mathematical model (Equation (1)) clearly showed that there was a linear and positive relationship between the response and both independent variables. This outcome indicated that the higher the treatment temperature and the longer the residence time, within the limits considered, the higher the polyphenol recovery.

On the other hand, the synergistic and quadratic effects of either variable were non-significant (*p* > 0.05). The desirability function (Figure 4B) was used to determine the optimal predicted values for both *T* and *t*, which were 90 °C and 240 min, respectively. Under this set of conditions, the predicted maximum response was 66.42 ± 3.23 mg CAE g^−1^ DM. In order to have an experimental confirmation of model validity, the predicted optimal conditions were employed to carry out three individual treatments and determine the response. The Y_TP_ value calculated was 67.38 ± 2.26 mg CAE g^−1^ DM, which perfectly matched the predicted maximum response (*p* < 0.05). This result demonstrated that the model constructed may be used for credible predictions.

The level of OLL polyphenol recovery achieved with the methodology developed herein is comparable to 69.35 mg GAE g^−1^ DM, reported for an organosolv OLL process using a glycerol/citric acid deep eutectic solvent [38]. Other recent examinations with acidified and/or hydroethanolic solvents attained values of 183.4 [39], 157.62 [31], 86.4 [34], and 76.1 mg GAE g^−1^ DM [32]. However, it is to be emphasized that large variations may be observed in Y_TP_, depending on the extraction technology utilized, i.e., conventional or microwave- or ultrasound-assisted extraction [31], and Y_TP_ may range from 7.35 to 138.4 mg GAE g^−1^ DM [40] and from 20.9 to 144.2 mg GAE g^−1^ DM [41]. Moreover, it should be noted that the above-mentioned values are merely indicative, since the actual total polyphenol yield does not depend solely on the extraction methodology, but also other important determinants, such as the olive variety and the seasonal variations. Such parameters have been shown to be the source of wide differences found for the polyphenolic content of OLLs [42,43,44].

### 2.5. Effect of Treatments on Extract Composition

In the light of a recent study that demonstrated extensive alteration in the OLL polyphenolic profile as a result of solvent effects on certain substances [38], liquid chromatography-mass spectrometry analyses were undertaken to detect possible such effects exerted by the treatments used. Target ions corresponding to molecular ions [M − H]^−^ of major OLL metabolites, including hydroxytyrosol derivatives (oleuropein and isomers thereof), and luteolin and apigenin derivatives (glycosides), were selected to trace changes originating from the different treatments performed.

**Figure 6:**
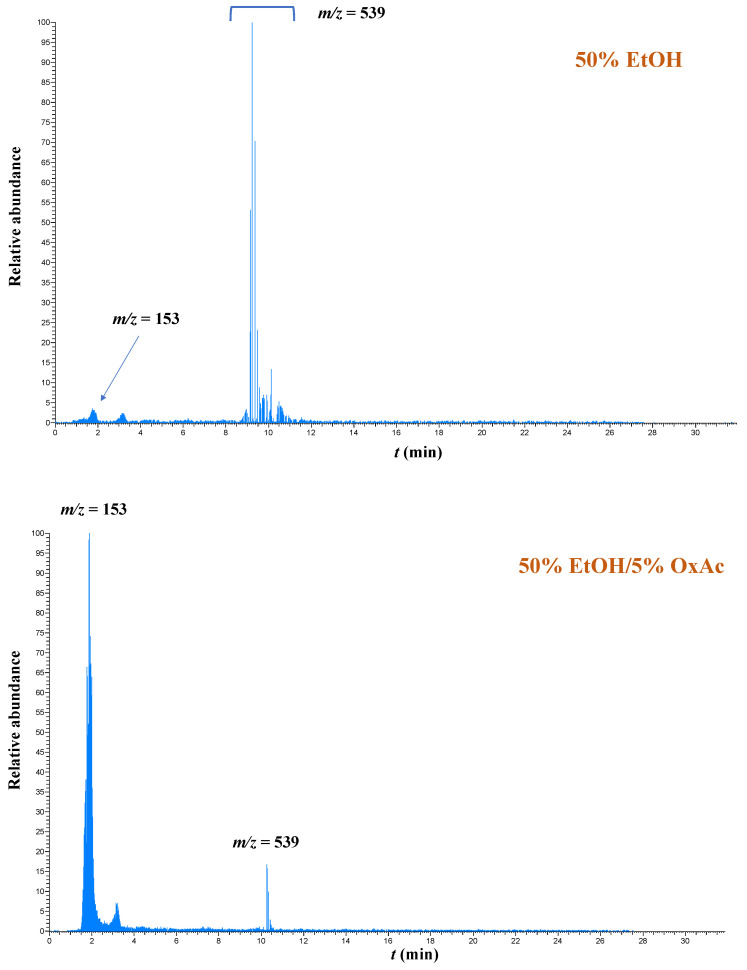
Selected ion chromatograms illustrating the transformation of oleuropein and its isomers after OLL treatment with 50% ethanol/5% oxalic acid.

**Figure 7:**
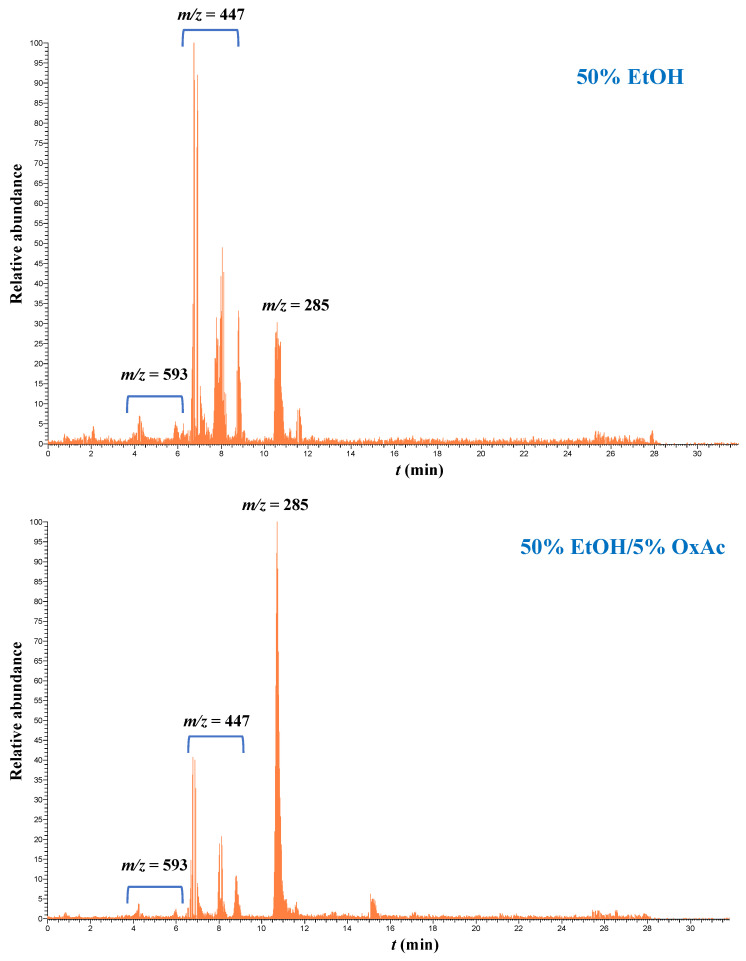
Selected ion chromatograms illustrating the transformation of luteolin glycosides after OLL treatment with 50% ethanol/5% oxalic acid.

With regard to oleuropein and its derivatives, they were detected by selecting the molecular ion [M − H]^−^ with *m*/*z* = 539.1 [45,46], and it can be seen in Figure 6 that they were dominant in extracts obtained with 50% ethanol (EtOH), while hydroxytyrosol was also detectable (molecular ion [M − H]^−^ with *m*/*z* = 153.2). Upon treatment with 50% ethanol/5% OxAc, the peaks corresponding to the molecular ion [M − H]^−^ with *m*/*z* = 539.1 virtually disappeared, whereas by far the dominant peak was the one corresponding to hydroxytyrosol. Likewise, peaks with a molecular ion [M − H]^−^ of *m*/*z* = 447.3 which correspond to luteolin hexosides were the principal compounds in the hydroethanolic extracts, accompanied by luteolin rutinosides (molecular ion [M − H]^−^ with *m*/*z* = 593.1) and the luteolin aglycone (molecular ion [M − H]^−^ with *m*/*z* = 285.1) (Figure 7).

**Figure 8:**
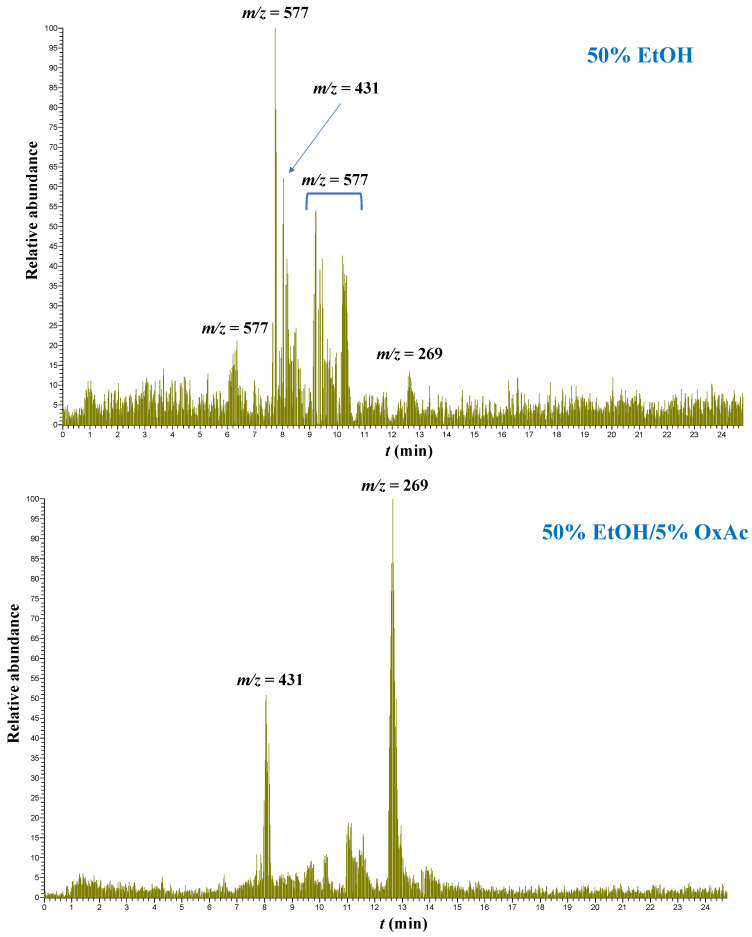
Selected ion chromatograms illustrating the transformation of apigenin glycosides after OLL treatment with 50% ethanol/5% oxalic acid.

However, in hydroethanolic extracts containing OxAc, the major compound was aglycone (Figure 7). A similar pattern was observed for apigenin and its derivatives, and in the hydroethanolic extracts apigenin rutinosides (*m*/*z* = 577) and a hexoside (*m*/*z* = 431) were the most abundant derivatives. On the other hand, the presence of OxAc afforded extracts enriched in the aglycone apigenin (Figure 8).

All these modifications were also seen in the extracts generated with HCl instead of OxAc. Thus, to gain a deeper insight into the changes that occurred as a result of the acid-catalyzed treatments, the release of hydroxytyrosol, luteolin, and apigenin, which represent the complete hydrolysis products, was quantified in all extracts produced.

In Table 3, it is illustrated that in the extracts obtained with the hydrothermal treatment, hydroxytyrosol, luteolin, and apigenin occurred at very low levels. However, the HCl-catalyzed treatment afforded a hydroxytyrosol yield of almost 12.7 mg g^−1^ DM, whereas the yields of both luteolin and apigenin were negatively affected. On the contrary, when hydrothermal treatment was carried out in the presence of OxAc instead of HCl, hydroxytyrosol liberation was boosted at approximately 20.2 mg g^−1^ DM and that of luteolin at 0.12 mg g^−1^ DM. Once again, the apigenin yield was lower in the presence of an acid catalyst.

The image depicted by applying the organosolv treatment was fundamentally different. First, it was observed that treatment with a neat hydroethanolic mixture afforded much higher yields for all three compounds (hydroxytyrosol, luteolin, apigenin) considered. This finding pointed to the increased dissolution of these polyphenols in ethanol/water mixtures than in water. Furthermore, the HCl-catalyzed treatment outperformed all other treatments, giving the highest yields for hydroxytyrosol, luteolin, and apigenin, although the effect on the latter was negligible. By contrast, the OxAc-catalyzed treatment provided significantly lower yields in both hydroxytyrosol and luteolin (*p* < 0.05), yet once again apigenin was not affected to a significant extent.

Oleuropein, and also flavone (luteolin and apigenin) glycosides occurring in OLL, may be prone to hydrolytic reactions, depending on the solvent used. Such a phenomenon has been observed during OLL polyphenol extraction using deep eutectic solvents, where evidence was raised of hydroxytyrosol generation at the expense of oleureropein [47]. Latter studies demonstrated that luteolin aglycone may appear in OLL extracts treated with slightly alkaline deep eutectic solvents [38]. In general, acid-catalyzed hydrolysis has been an efficient means of liberating flavonoid aglycones from their corresponding glycosides [48]. Yet, hydrolysis of flavonoid glycosides may also occur at a near neutral pH upon hydrothermal treatment [49], while high processing temperatures (130–165 °C) may also promote efficient flavonoid glycoside hydrolysis [50]. On the other hand, the use of a weak organic acid, such as OA used herein, has never been previously reported to catalyze relevant reactions. This novel finding highlighted for the first time the usefulness of OxAc in performing polyphenol glycoside hydrolysis, using either hydrothermal or organosolv processes.

### 2.6. Treatment Effect on Antioxidant Activity

**Figure 9:**
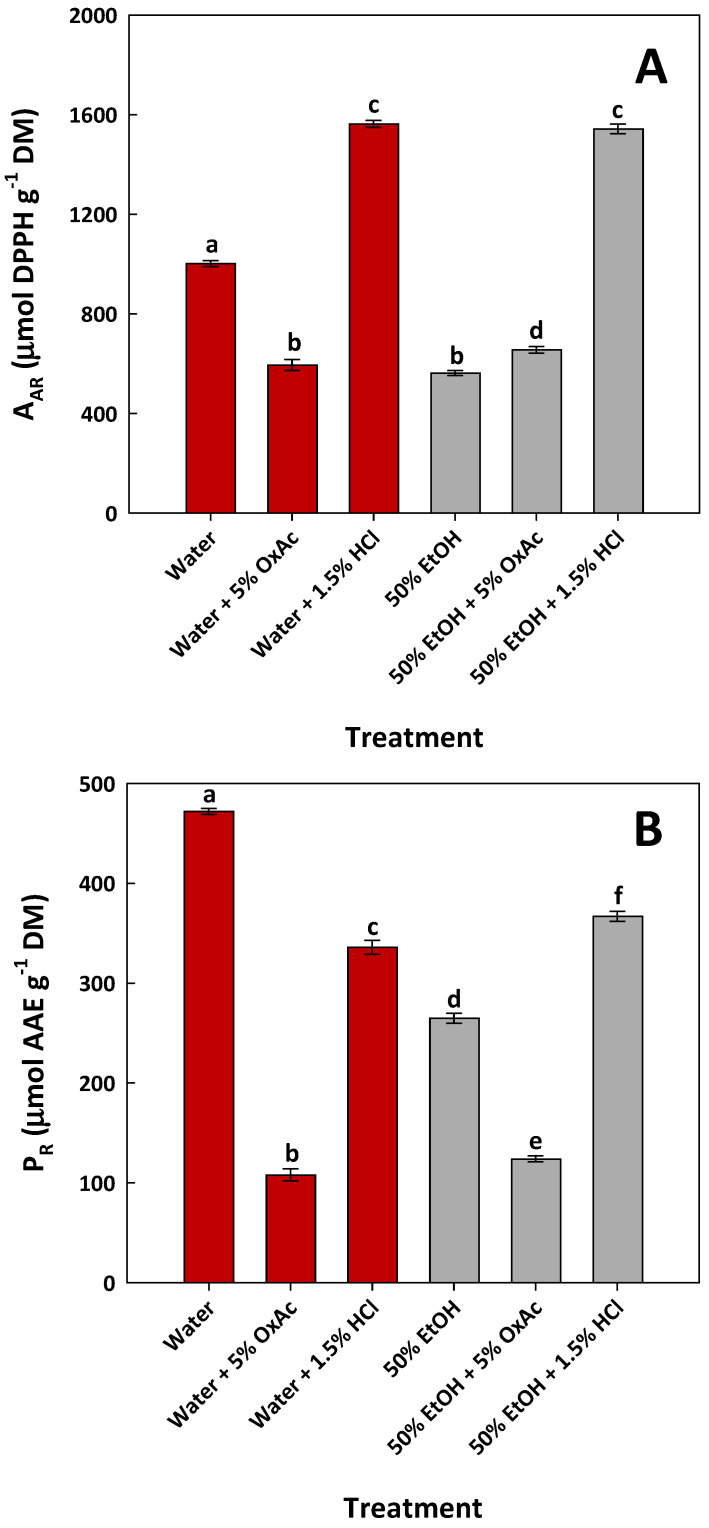
Bar plots showing the antiradical activity (**A**) and the ferric-reducing power (**B**) of the extracts obtained by treating OLLs with various solvent systems, under optimized conditions. Bars assigned with different letters (a–f) represent statistically different values (*p* < 0.05).

The antioxidant activity was evaluated to detect the possible impact of the compositional changes on the antioxidant characteristics of the extracts, given the drastic modifications found in the polyphenolic composition of the extracts as a result of the acid-catalyzed treatments. To this end, antiradical activity (A_AR_) and ferric-reducing power (P_R_) were determined, and the results are illustrated in Figure 9.

Concerning A_AR_, extracts from both HCl-catalyzed hydrothermal and organosolv treatments exhibited an equal performance, which was by far higher than all the other extracts assayed. On the contrary, the highest P_R_ was displayed by the aqueous extract, which outperformed all other extracts. This outcome suggested that the modifications in the composition recorded could not sufficiently justify the antioxidant behavior, as revealed by the two tests performed. Regarding A_AR_, it could be argued that the increased levels of both hydroxytyrosol and luteolin in the 50% EtOH/1.5% HCl extract might have a significant contribution, but this could not explain the similar A_AR_ value found for the water/1.5% HCl extract. Moreover, the extracts obtained through the water/5% OxAc and 50% EtOH/5% OxAc treatment exhibited a consistently low A_AR_, although they both contained increased hydroxytyrosol levels. On the other hand, P_R_ did not seem to be influenced by either treatment, since the extract produced with near water displayed the highest reducing effect.

The interpretation of the results from antioxidant tests on OLL extracts is a particularly complicated task, given the outstanding complexity related to the expression of antioxidant activity by the major OLL constituents [51]. Early studies evidenced the correlation of total polyphenol concentration with antiradical effects [52], which has also been supported by more recent studies [32,43], yet other investigations emphasized the contribution of the flavonoid fraction [53] and specific metabolites, such as luteolin 7-O-glucoside [54] and oleuropein [55]. In addition, a number of OLL substances showed a positive and strong correlation with both antiradical and ferric-reducing effects, most of them being oleuropein derivatives [33].

## 3. Materials and Methods

### 3.1. Chemicals

Hydroxytyrosol (98%), oleuropein (98%), luteolin 7-O-glucoside (>98%), apigenin (≥95%), ascorbic acid, 2,2-diphenylpicrylhydrazyl (DPPH), tartaric acid (>99.7%), oxalic acid (98%), and caffeic acid were from Sigma-Aldrich (Darmstadt, Germany). Iron(III) chloride hexahydrate, sodium carbonate (>99.8%), and 2,4,6-tripyridyl-s-triazine (TPTZ) were from Honeywell/Fluka (Steinheim, Germany). Ethanol was from Honeywell/Riedel-de Haen (Seelze, Germany). Folin–Ciocalteu reagent and citric acid (99%) were from Merck (Darmstadt, Germany). Solvents of appropriate purity (HPLC grade) were used for chromatographic analyses.

### 3.2. Collection and Processing of Olive Leaves (OLLs)

Fresh OLLs, with no apparent infections or damages, were collected in October 2023 from an olive tree plantation located within the premises of M.A.I.Ch. (latitude: 35 29 42.291600 north; longitude: 24 02 53.988000 west). The plantation embraces trees of the Koroneiki variety (*O. europaea*) and receives neither watering nor fertilizing. The collection was accomplished by gathering leaves from various trees and from different parts of each tree (sun exposed, shaded), which were then pooled to form a total amount of about 1 kg. OLLs were then dried in a laboratory oven at 100 °C, for 2 h, and comminuted and sieved to provide a powder with <300 μm mean particle diameter. This material was used for all further treatments.

### 3.3. Screening of Acid Catalyst

Three naturally occurring, food-grade organic acids were tested for their effectiveness in the recovery of OLL polyphenols, namely citric acid, oxalic acid, and tartaric acid. Hydrochloric acid (HCl) was used as a reference acid catalyst. To screen the effect of the different acids and various concentrations thereof, aqueous solutions of 5, 7.5, and 10% (*w*/*v*) were tested. HCl was tested at a constant concentration of 1.5%. Each aqueous acid solution (50 mL) was combined with 2.5 g of OLLs in a 100 mL Duran™ bottle (DWK Life Sciences, Wertheim, Germany), and treated for 180 min, at 90 °C, under a constant agitation speed of 500 rounds per minute (rpm). Both heating and agitation were provided by an oil bath placed on a hotplate/magnetic stirrer (AREC.X, Velp Scientifica, Usmate, Italy). After treatment, each extract was allowed to acquire ambient temperature and centrifuged at 11,500× *g* for 10 min to separate cell debris.

### 3.4. Hydrothermal Treatment

OLLs were treated with aqueous solutions of 1.5% HCl and 5% oxalic acid, using various combinations of residence time (10–240 min) and temperature (50–90 °C). All other conditions and sample handling were as described in Section 3.3.

### 3.5. Ethanol Organosolv Treatment

OLLs were treated with solutions of 1.5% HCl and 5% oxalic acid containing ethanol, whose concentration varied from 10 to 70% (*v*/*v*), using various combinations of residence time (10–240 min) and temperature (50–90 °C). All other conditions and sample handling were as described in Section 3.3.

### 3.6. Extraction Kinetics—The Effect of Temperature

Kinetics was traced using a previously proposed first-order model [56]:

Y_TP(*t*)_ = Y_TP(*s*)_(1 − *e^−kt^*) (2)

where Y_TP(*t*)_ is the total polyphenol yield at any time *t*, Y_TP(*s*)_ the total polyphenol yield at saturation (equilibrium), and *k* the first-order extraction rate constant (min^−1^). Process activation energy (*E*_a_) was determined as follows [57]:

(3) $$ln(\frac{k}{k_{ref}})=(-\frac{E_{a}}{R})(\frac{1}{T}-\frac{1}{T_{ref}})$$

*T*_ref_ and *T*, both given in K, represent the reference temperature (70 °C in this study) and the temperature at which the kinetics was traced, respectively. The terms *k*_ref_ and *k* are the corresponding second-order extraction rate constants, *R* the universal gas constant (8.314 J K^−1^ mol^−1^), and *E*_a_ the activation energy (J mol^−1^).

### 3.7. Treatment Optimization through Response-Surface Methodology

To assess the effect of the two key process variables, temperature (*T*) and residence time (*t*), a Box–Behnken experimental design with three central points was deployed to come up with a predictive model, using response-surface methodology. Considering the outcome from the single-factor testing, the actual levels of both T and t were codified in three levels, −1, 0, and 1, as previously described [58]. Details are given in Table 4.

The constructed model was evaluated on the basis of both analysis of variance (ANOVA) and lack-of-fit tests, which permitted the determination of the overall model significance and also the significance of each model term. The predictive model was given as a mathematical equation containing only significant terms, whereas the non-significant ones (*p* > 0.05) were not considered.

### 3.8. Analytical Determinations

To determine the total polyphenol concentration in the extracts produced, a previously published protocol was followed, based on the Folin–Ciocalteu methodology [58]. The results were calculated as mg caffeic acid equivalents (CAE), using a gallic acid standard curve, and the yield in total polyphenols was expressed as mg GAE per g dry OLL mass (DM). The ability of extracts to reduce Fe^3+^ was determined using a ferric-reducing power (P_R_) test, based on the chromophore complexing agent TPTZ [58]. The results were given as μmol ascorbic acid equivalents (AAE) per g DM. The antiradical activity (A_AR_) was estimated using the stable radical DPPH and given as μmol DPPH per g DM [58]. In all spectrophotometric determinations, suitable blanks were used for quantification.

### 3.9. Liquid Chromatography–Diode Array-Tandem Mass Spectrometry (LC-DAD-MS)

All samples were filtered through 0.45 μm syringe filters (Tisch Scientific, Cleves, OH, USA) prior to determinations. The equipment employed was a TSQ Quantum Access LC/MS/MS, with a Surveyor pump (Thermo Scientific, Walltham, MA, USA), and an Acquity PDA detector (Waters, Milford, MT, USA) interfaced by XCalibur 2.1, TSQ 2.1 software. Chromatographic determinations were accomplished with a Fortis RP-18 column (150 mm × 2.1 mm, 3 μm) at 40 °C, using a 10 μL injection volume and a flow rate of 0.3 mL min^−1^. Eluent A was 1% aqueous acetic and eluent B was 99% acetonitrile/1% acetic acid. The elution program applied was as follows: 0–2 min, 5% B; 2–27 min, 50% B; 27–29 min, 100% B. The settings used for acquiring mass spectra in negative ionization mode, were the following: spray voltage, 3000 V, sheath gas pressure, 30 (arbitrary units), auxiliary gas pressure, 10 (arbitrary units), and capillary temperature, 350 °C. Quantification was carried out using the peak areas obtained with UV–Vis detection at 280 and 340 nm, for the hydroxytyrosol, and luteolin and apigenin, respectively, with external standard methodology. Calibration curves were established with hydroxytyrosol (R^2^ = 0.9987), luteolin 7-O-glucoside (for luteolin quantification) (R^2^ = 0.9990), and apigenin (R^2^ = 0.9991). The curves were constructed using standards with concentrations 0–50 μg mL^−1^, freshly prepared in methanol.

### 3.10. Statistical Handling

The design of the experiment, response-surface methodology deployment, and relevant statistics (ANOVA, lack-of-fit) were computed with JMP™ Pro 16 (SAS, Cary, NC, USA). Non-linear and linear regressions (at least 95% significance level) were established with SigmaPlot™ 15.0 (Systat Software Inc., San Jose, CA, USA). Data were not found to be normally distributed by performing the Shapiro–Wilk test, and thus statistically significant differences were ascertained by the Kruskal–Wallis test, using IBM SPSS Statistics™ 29 (SPSS Inc., Chicago, IL, USA). Single-factor screening and organosolv treatments were performed at least twice, and all analyses (spectrophotometric, chromatographic) were conducted in triplicate. Values were given as the average ± standard deviation (SD).

## 4. Conclusions

The acid-catalyzed treatment of olive leaves using both strong (HCl) and mild (oxalic acid) catalysts afforded extracts enriched in polyphenols arising from the hydrolysis of native olive leaf constituents, such as oleuropein and flavonoid glycosides. The evidence emerged suggested that there was differentiated behavior of each catalyst in aqueous and hydroethanolic environments; yet, under specific conditions, extensive hydrolysis may be achieved. In any case, the major substance determined in the extracts produced was hydroxytyrosol, which might be associated with the enhanced antioxidant activity observed. Considering that oleuropein and flavone glycosides are not suitable antioxidants for foods such as bulk oil, due to their high polarity, a methodology that would combine concomitant hydrolysis and recovery would afford extracts enriched in phenolics that could be easily incorporated into fatty material. Although one limitation that might arise by such a transformation would be the alteration of the biological properties of these phytochemicals, this prospect is of particular importance in waste biomass valorization, paving new pathways for the production of high value-added commodities, such as natural food antioxidants. Thus, the treatments proposed may be well integrated into larger biorefinery processes, contributing to fully harnessing materials rejected from the food industry. In this view, sustainable strategies may enable technologies that would provoke less environmental aggravation and the production of foods fortified with natural functional constituents.

## Figures and Tables

**Table 1:** Kinetic data and energetic barriers determined by implementing first-order kinetics for the polyphenol recovery from OLLs, using various solvent systems.

	Treatment	*T* (°C)	R^2^ *	*k* (min^−1^)	Y_TP(s)_ (mg CAE g^−1^ DM)	*E*_a_ (kJ mol^−1^)
**Hydrothermal treatment**	Water	50	0.93	0.0398	37.96 ^a^	0.52
		70	0.92	0.0401	34.26 ^b^	
		90	0.97	0.0405	41.97 ^a^	
	Water + 1.5% HCl	50	0.95	0.0417	47.37 ^c^	5.88
		70	0.98	0.0653	48.94 ^c^	
		90	0.99	0.0731	49.06 ^c^	
	Water + 5% OxAc	50	0.93	0.0535	41.05 ^a^	5.02
		70	0.99	0.0751	45.01 ^d^	
		90	0.99	0.0827	47.33 ^c^	
**Organosolv treatment**	50% EtOH	50	0.97	0.0334	33.79 ^b^	19.80
		70	0.99	0.0372	36.27 ^b^	
		90	1.00	0.0544	39.62 ^a^	
	50% EtOH + 1.5% HCl	50	0.99	0.0219	42.94 ^a^	32.81
		70	0.99	0.0261	47.81 ^c^	
		90	0.99	0.0490	49.32 ^c^	
	50% EtOH + 5% OxAc	50	0.96	0.0143	59.10 ^e^	12.84
		70	0.96	0.0186	61.27 ^e^	
		90	0.98	0.0238	65.36 ^f^	

* Indicates correlation of the kinetic model fitting to the experimental data. Values denoted with different letters (a–f) are statistically different (*p* < 0.05).

**Table 2:** Illustration of the measured (actual) and predicted response values, after deploying response-surface methodology.

Design Point	Independent Variables		Responses	
			Y_TP_ (mg CAE g^−1^ DM)	
	X_1_ (*T*, °C)	X_2_ (*t*, min)	Measured	Predicted
1	−1 (120)	−1 (50)	47.97	48.69
2	−1 (120)	1 (90)	58.35	57.35
3	1 (240)	−1 (50)	59.55	60.31
4	1 (240)	1 (90)	67.38	66.42
5	−1 (120)	0 (70)	52.74	53.02
6	1 (240)	0 (70)	63.16	63.36
7	0 (180)	−1 (50)	56.04	54.52
8	0 (180)	1 (90)	59.93	61.90
9	0 (180)	0 (70)	58.40	58.21
10	0 (180)	0 (70)	58.92	58.21
11	0 (180)	0 (70)	57.83	58.21

**Table 3:** Changes in hydroxytyrosol, luteolin and apigenin recorded after treatment of OLLs with the various solvent systems.

Treatment	Y (mg g^−1^ DM)		
	Hydroxytyrosol	Luteolin	Apigenin
Water	0.071 ± 0.05 ^a^	0.05 ± 0.01 ^a^	0.09 ± 0.01 ^a^
Water + 1.5% HCl	12.7 ± 0.8 ^b^	0.03 ± 0.01 ^b^	0.02 ± 0.00 ^b^
Water + 5% OxAc	20.2 ± 0.9 ^c^	0.12 ± 0.01 ^c^	0.04 ± 0.00 ^c^
50% EtOH	0.63 ± 0.05 ^d^	0.12 ± 0.01 ^c^	0.02 ± 0.00 ^b^
50% EtOH + 1.5% HCl	23.4 ± 0.8 ^e^	1.96 ± 0.08 ^d^	0.02 ± 0.00 ^b^
50% EtOH + 5% OxAc	10.3 ± 0.6 ^f^	0.72 ± 0.02 ^e^	0.02 ± 0.01 ^b^

Values designated with different letters within columns are statistically different (*p* < 0.05).

**Table 4:** The process variables considered in this study and their coded and actual values.

Variable	Code	Levels		
		−1	0	1
*T* (°C)	X_1_	50	70	90
*t* (min)	X_2_	120	180	240

## Data Availability

Data are contained within the article.
